# Silver nanoparticles enhance the efficacy of aminoglycosides against antibiotic-resistant bacteria

**DOI:** 10.3389/fmicb.2022.1064095

**Published:** 2023-01-31

**Authors:** Autumn S. Dove, Dominika I. Dzurny, Wren R. Dees, Nan Qin, Carmen C. Nunez Rodriguez, Lauren A. Alt, Garrett L. Ellward, Jacob A. Best, Nicholas G. Rudawski, Kotaro Fujii, Daniel M. Czyż

**Affiliations:** ^1^Department of Microbiology and Cell Science, College of Agricultural and Life Sciences, University of Florida, Gainesville, FL, United States; ^2^Natural Immunogenics Corporation, Sarasota, FL, United States; ^3^Research Service Centers, Herbert Wertheim College of Engineering, University of Florida, Gainesville, FL, United States; ^4^Department of Molecular Genetics and Microbiology, College of Medicine, University of Florida, Gainesville, FL, United States; ^5^Center for NeuroGenetics, University of Florida, Gainesville, FL, United States

**Keywords:** silver nanoparticles, antibiotic resistance, *Caenorhabditis elegans*, antimicrobials, synergism, antibiotic potentiators, aminoglycosides

## Abstract

As the threat of antimicrobial-resistant bacteria compromises the safety and efficacy of modern healthcare practices, the search for effective treatments is more urgent than ever. For centuries, silver (Ag) has been known to have antibacterial properties and, over the past two decades, Ag-based nanoparticles have gained traction as potential antimicrobials. The antibacterial efficacy of Ag varies with structure, size, and concentration. In the present study, we examined Ag nanoparticles (AgNPs) for their antimicrobial activity and safety. We compared different commercially-available AgNPs against gram-negative *Escherichia coli*, *Pseudomonas aeruginosa*, *Acinetobacter baumannii*, and gram-positive *Staphylococcus aureus* methicillin-resistant and susceptible strains. The most effective formula of AgNPs tested had single-digit (μg/mL) minimum inhibitory concentrations against gram-negative multidrug-resistant clinical bacterial isolates with novel and emerging mechanisms of resistance. The mode of killing was assessed in *E. coli* and was found to be bactericidal, which is consistent with previous studies using other AgNP formulations. We evaluated cytotoxicity by measuring physiological readouts using the *Caenorhabditis elegans* model and found that motility was affected, but not the lifespan. Furthermore, we found that at their antibacterial concentrations, AgNPs were non-cytotoxic to any of the mammalian cell lines tested, including macrophages, stem cells, and epithelial cells. More interestingly, our experiments revealed synergy with clinically relevant antibiotics. We found that a non-toxic and non-effective concentration of AgNPs reduced the minimum inhibitory concentrations of aminoglycoside by approximately 22-fold. Because both aminoglycosides and Ag are known to target the bacterial ribosome, we tested whether Ag could also target eukaryotic ribosomes. We measured the rate of mistranslation at bactericidal concentration and found no effect, indicating that AgNPs are not proteotoxic to the host at the tested concentrations. Collectively, our results suggest that AgNPs could have a promising clinical application as a potential stand-alone therapy or antibiotic adjuvants.

## 1. Introduction

The misuse of antibiotics across One Health sectors fuels an irreversible increase in resistance and renders clinically important antibiotics ineffective. Over 2.8 million antibiotic-resistant infections occur annually in the United States, leading to more than 35,000 deaths (CDC, 2019). A recent report estimates that globally, 1.27 million deaths were directly attributed to antimicrobial resistance (AMR) in 2019 (Murray et al., 2022). In addition to the immense cost to human life, AMR also leaves a global economic burden. The total annual economic losses in the United States have been as high as $20 billion in health care and $35 billion in productivity (CDC, 2013). According to the 2019 CDC AMR report, some of the urgent threats to human health include carbapenem-resistant *Acinetobacter baumannii* and carbapenem-resistant *Enterobacterales*; while some of the serious threats include extended-spectrum β-lactamase-producing *Enterobacteriaceae*, multidrug-resistant (MDR) *Pseudomonas aeruginosa*, and methicillin-resistant *Staphylococcus aureus* (MRSA) (CDC, 2019). All the pathogens listed can cause skin and soft tissue infections and are resistant to many commonly used antimicrobials. For example, *Escherichia coli* can cause necrotizing soft tissue infections (Bayard et al., 2021), *P. aeruginosa* is associated with burn wounds (Wu et al., 2011), both methicillin-resistant and sensitive strains of *S. aureus* are associated with hospital and community-acquired skin infections (Moffarah et al., 2016), and *A. baumannii* can cause skin and soft tissue infections, commonly seen in combat soldiers deployed to the Middle East (Sebeny et al., 2008). *E. coli*, *S. aureus*, *K. pneumoniae*, *Streptococcus pneumoniae*, *P. aeruginosa*, and *A. baumannii* are among the most prevalent AMR pathogens, collectively contributing to over 80% of all AMR-related deaths (Murray et al., 2022). Most of these bacteria are commonly referred to as the ESKAPE pathogens (Boucher et al., 2009). The availability of effective therapies is further limited by the lack of development of new antibiotics. Novel non-traditional antimicrobial approaches are currently being explored as potential therapeutics. Some of the most promising strategies include bacteriophage therapy (Kakasis and Panitsa, 2019), drug repurposing (Miró-Canturri et al., 2019), and silver nanoparticles (AgNPs) (Rai et al., 2009; Bruna et al., 2021).

For centuries, Ag has been known to have antimicrobial properties. It has been used for many purposes, including patented medical-grade products containing Ag as an antimicrobial agent (Sim et al., 2018). The expansion of more therapeutic options, like antibiotics in the 1940s, led to a decrease in the utilization of Ag for antimicrobial purposes. One of the first studies that led to a resurgence of interest in Ag used 0.5% Ag nitrate to treat burn wounds (Moyer et al., 1965). The current dearth of effective antibiotics has forced physicians to reach for archaic antibiotics with potentially life-threatening adverse effects (Falagas and Kasiakou, 2005). Therefore, it is not surprising that other therapeutics, including Ag, are re-emerging as antimicrobial candidates (Franci et al., 2015). The recent advancements in nanotechnology leading to the development and characterization of AgNPs have significantly broadened the potential application of Ag. Numerous studies demonstrated the antimicrobial property of AgNPs against a variety of antibiotic-resistant bacteria, including MRSA, erythromycin-resistant *Streptococcus pyogenes*, ampicillin-resistant *E. coli*, MDR *P. aeruginosa*, *Listeria monocytogenes*, *Klebsiella pneumoniae*, and *K. aerogenes* (Lara et al., 2010; Brown et al., 2012; Bose and Chatterjee, 2016; Mohamed et al., 2020; Campo-Beleño et al., 2022). Although the exact mechanism of their antimicrobial property is not known, various reports suggest that they target the bacterial cell membrane (Jung et al., 2008; Bondarenko et al., 2018) and the ribosome and protein synthesis (Yamanaka et al., 2005). There is also evidence that AgNPs increase the generation of reactive oxygen species, leading to oxidative stress and, ultimately, DNA and protein damage (Park et al., 2009). Furthermore, AgNPs were shown to be involved in the disruption of cellular signal transduction pathways (Shrivastava et al., 2007). The size and shape of AgNPs affect their antibacterial property, with smaller spherical and triangular particles having the highest efficacy (Pal et al., 2007; Raza et al., 2016; Dong et al., 2019). It was shown that a size decrease from ~11 to ~3 nm led to an enhancement of AgNP antibacterial activity, and particles between 1 and 10 nm had optimal antibacterial properties (Morones et al., 2005; Bin Ahmad et al., 2012). In one study, it was shown that a membrane of chitosan, gelatin, polyvinyl pyrrolidone and AgNPs incubated with bacteria led to reduced expression of *mecA* and *erm* resistance genes in *S. aureus* and *Bacillus subtilis*, and β-lactamase and *erm* in *E. coli* and *Salmonella typhi*. Over-expression of these genes induces tolerance to antibiotics; hence, this membrane was developed as a potential wound dressing supplemented with AgNPs to help prevent MDR infections in burn wounds and other AMR skin infections (El-Aassar et al., 2021). AgNPs that showed antibacterial activity against *E. coli* and *S. aureus in vitro* also promoted faster wound healing in a mouse model (Chinnasamy et al., 2021). These results have been corroborated by other studies that demonstrated the wound-healing properties of AgNPs and their antimicrobial activity against methicillin-resistant *Staphylococcus pseudintermedius* wound infection (Thammawithan et al., 2021; Zhou et al., 2021). AgNPs were also shown to enhance the effectiveness of several antibiotics, including amoxicillin, penicillin G, erythromycin, vancomycin, clindamycin, imipenem, and cefixime (Shahverdi et al., 2007; Naqvi et al., 2013; Ali et al., 2020); however, the underlying mechanisms remain unknown.

In the present study, we evaluate the efficacy and toxicity of AgNPs as a potential antimicrobial against a broad spectrum of bacteria, including those with novel mechanisms of resistance. Additionally, the direct bactericidal effects and synergy of AgNPs with aminoglycoside antibiotics, as seen with a 22-fold reduction in minimum inhibitory concentration (MIC) of amikacin, suggest a promising utility of AgNPs as antibiotic adjuvants. Furthermore, the lack of cytotoxicity to cells, no effect on *C. elegans* lifespan, and no proteotoxic property of AgNPs support their use for broad antimicrobial applications.

## 2. Materials and methods

### 2.1. Strains, chemicals, and culture materials

#### 2.1.1. Bacterial strains and culture

Bacterial strains used in this study include *E. coli*, *P. aeruginosa, S. aureus*, *A. baumannii*, *Salmonella* spp., *K. pneumoniae*, and *Citrobacter freundii*. More detailed information about each strain can be found in Supplementary Table S1. Briefly, the clinical bacterial strains are MDR and carry novel resistance mechanism. The strains are made available through the Antibiotic Resistance Isolate Bank collection maintained by the Centers for Disease and Control and Prevention (CDC) and the Food and Drug Administration (FDA). All bacteria were cultured at 37°C, shaking at 220 rpm. *S. aureus* and MRSA were cultured in Tryptic soy broth (TSB). All other strains were cultured in lysogeny broth (LB) Lennox.

#### 2.1.2. Tissue culture

Cell lines used in this study are found in Supplementary Table S1. THP-1 cells were maintained in Roswell Park Memorial Institute 1640 medium (RPMI 1640) supplemented with 10% fetal bovine serum (FBS). Differentiation of THP-1 cells from monocytes to macrophages was performed by adding 40 ng/mL phorbol-12-myristate-13 acetate (PMA) to the cell medium and incubating for 72 h. RAW 264.7 macrophages and HeLa cells were maintained in Dulbecco’s Modified Eagle Medium (DMEM), supplemented with 10% FBS. The mouse embryonic stem cells (mESCs) were maintained in knockout DMEM supplemented with 1x L-glutamine, 1x non-essential amino acids, 0.1% 2-mercaptoethanol, leukemia inhibiting factor, 15% ESC grade FBS, and 1x ES grade penicillin/streptomycin. All cell experiments were performed at 37°C with 5% CO_2_ in a humidified environment.

#### 2.1.3. *Caenorhabditis elegans* culture

All experiments were performed at 25°C using the N2 wild-type strain (Supplementary Table S1). Nematodes were maintained on a nematode growth medium (NGM) according to previously established protocols (Brenner, 1974).

#### 2.1.4. Chemicals

Chemicals and reagents are listed in Supplementary Table S1. AgNPs were manufactured and supplied by Natural Immunogenics Corporation. Two types of AgNP products (Formula 1 and 2) were tested, and five batches from each formula were used. Aztreonam, cefoxitin, doripenem, and trimethoprim were dissolved in dimethyl sulfoxide (DMSO). Tetracycline was dissolved in ethanol, while all other antibiotics were dissolved in water.

### 2.2. Characterization of AgNPs

Ag concentrations were measured by atomic absorption spectroscopy (AAS). One milliliter of the sample was digested in 1 mL of 70% nitric acid using a microwave and then diluted to 10 mL using DI water. Triplicate measurements were performed for each sample.

Transmission electron microscopy (TEM) samples were prepared using Formvar-C coated 300 mesh Cu grids. A 1.0 μl drop was deposited onto each grid and allowed to dry in a desiccator. Bright-field transmission electron microscopy (BF-TEM) imaging was performed using a Phillips CM100 TEM operated at 100 kV to analyze the particle size distribution. Two grids were imaged, and a minimum of three images per grid were used to obtain average particle size data. The particle outlines were identified using ImageJ software. Images were visually inspected for artifacts to ensure the maximum inclusion of actual particles. The particle diameters were calculated from the outlined areas assuming a circular shape.

Additionally, atomic-resolution high-angle annular dark-field scanning transmission electron microscopy (HAADF-STEM) imaging was performed using an FEI Themis scanning TEM (S/TEM) with C_S_ probe correction operated at 200 kV. This allowed direct visualization of the atomic structures of the AgNPs. Immediately prior to HAADF-STEM imaging, each grid was plasma cleaned for a few seconds using a Fischione Instruments Model 1,020 plasma cleaner. This was required for successful atomic-resolution HAADF-STEM imaging to occur; otherwise, the build-up of carbonaceous contamination during imaging prohibited this.

### 2.3. Assessing minimum inhibitory concentrations and the antibacterial mode of inhibition of AgNPs

Serial dilutions of AgNPs were performed starting with 100 μl of undiluted stock added to the first row of a 96-well plate with 50 μl of LB per well in subsequent rows. An overnight culture was grown as described above; the optical density (OD_600_) was adjusted to 1.0, then diluted 1:500 in LB media, followed by aliquoting 50 μl to each well, resulting in a final dilution of 1:1000. All concentrations of AgNPs were ultimately diluted by half as a result of the addition of the bacteria to each well, leaving the highest concentration as half of the stock. The plate was incubated at 37°C in the Tecan Infinite 200 Pro plate reader for 20 h. Every 30 min, the plate was shaken orbitally for 5 s, and the OD_600_ readings were taken. The minimum inhibitory concentration (MIC) was defined as the lowest concentration that inhibited the growth of bacteria, as determined by a < 0.1 increase of OD_600_ over 20 h. The bactericidal or bacteriostatic activity of AgNPs was determined according to established protocols (Pankey and Sabath, 2004). To determine the minimum bactericidal concentration (MBC), after the 20 h reading, the culture was serially diluted and plated on LB agar. Colony-forming units (CFUs) were counted to determine the MBC. The ratio of the MBC to the MIC was found to be <4, indicating a bactericidal mode of inhibition. Experiments were performed with three independent runs of three replicates per run.

#### 2.3.1. Checkerboard assay

Serial dilutions of amikacin were performed in water in a 96-well plate starting with 100 μl of 64 μg/mL of amikacin in row A and diluted into 50 μl of water until row G. Only water was added to row H. In a separate plate, 200 μl of 12.4 μg/mL AgNPs was added to column 12 and then diluted in 100 μl of water from columns 11–2. Only water was added to column 1. From the AgNP plate, 50 μl was transferred and added to the amikacin dilution plate. A culture of *E. coli* 0494 was grown overnight as described above; the OD_600_ was adjusted to 1.0, then diluted 1:500 using 2x MHB. A volume of 100 μl was added to each well bringing the final volume to 200 μl per well, the highest concentration of amikacin to 16 μg/mL, the highest concentration of AgNPs to 3.1 μg/mL, and the bacteria to a dilution of 1:1000. To test the *E. coli* 0346 isolate this procedure was repeated with the highest concentration of amikacin starting at 800 μg/mL, bringing the final concentration to 200 μg/mL. The plate was incubated at 37°C in the Tecan Infinite 200 Pro plate reader for 20 h. The endpoint OD_600_ reading was used to determine the MIC. Experiments were performed with three independent runs. The interaction between AgNPs and amikacin was assessed by calculating the fractional inhibitory concentration index (FICI), where the FICI = FIC_AgNP_ + FIC_Amikacin_; FIC_AgNP_ = MIC_AgNP_ combination/MIC_AgNP_ alone; FIC_Amikacin_ = MIC_Amikacin_ combination/MIC_Amikacin_ alone. The FICI values indicate synergy (FICI ≤ 0.5), partial synergy (0.5<FICI ≤ 0.75), additive (0.75<FICI ≤ 1.0), indifferent (1.0 < FICI ≤ 4.0), or antagonistic (FICI>4.0) effect.

### 2.4. Cytotoxicity to host cells

Cell viability was measured using the CellTiter-Blue® cell viability assay. The assay was performed according to the manufacturer’s instructions (Promega). Briefly, THP-1 monocyte-derived macrophages were seeded into tissue culture-treated 96-well plates at a density of 5 × 10^4^ cells per well and differentiated into macrophages for 72 h in the presence of 40 ng/mL PMA. A 150 μl aliquot of the AgNP stock mixed with 150 μl of RPMI 1640 medium was added to the top row of a new 96-well plate, and 150 μL of culture medium was added to rows B-H, followed by 1:2 serial dilutions from row A through G, leaving H as the untreated control. The medium was aspirated from the wells containing cells, washed twice with phosphate-buffered saline (PBS), followed by a transfer of 100 μl of the diluted AgNPs or plain medium, and incubated for additional 24 h. Following the incubation, the medium was aspirated, cells were washed twice with PBS, and fresh medium and CellTiter-Blue® viability reagent were added at 100 and 20 μl volumes, respectively. The plate was incubated for an additional 24 h. The absorbance was measured via optical density at OD_570_ and OD_600_ in the Tecan Infinite 200 Pro microplate reader. The cytotoxicity was determined by subtracting the OD_570_ values (absorption maximum for resorufin) from the OD_600_ (absorption maximum for resazurin reference) values, normalizing the values to the untreated control, and calculating the percent of cells alive. The viability was also tested using HeLa cells, RAW 264.7 macrophages, and mESCs. Each cell line was treated with AgNPs for times specified for each of the experiments. All cell lines were tested with three independent runs. RAW 264.7 and THP1 macrophages were tested with two replicates per run, while HeLa and mESCs were tested with three replicates per run.

### 2.5. Bacterial uptake by macrophages

RAW 264.7 macrophages were seeded and incubated overnight as described above in tissue culture-treated 96-well plates at a density of 5 × 10^4^ cells/mL. The cells were allowed to attach overnight. AgNPs were serially diluted from stock concentrations as described above. Cells were washed twice with PBS, and AgNPs dilutions were transferred to the 96-well plate containing cells and incubated for 4 h. An overnight culture of *E. coli* DH5α was adjusted to an OD_600_ of 1.0 and diluted in DMEM. AgNP-containing medium was aspirated, and cells were washed twice with PBS. Bacteria were added to the cells at a multiplicity of infection (MOI) of 10 and the plates were centrifuged at 2200 RPMs for 30 min at 37°C, followed by a 30-min incubation at 37°C and 5% CO_2_. After the incubation, the well content was replaced with a fresh medium containing 100 μg/mL gentamicin to eliminate extracellular bacteria. The plates were incubated for 1 h. The medium was then aspirated, cells were washed twice with PBS, and lysed with 100 μl of 0.05% Triton X-100 for 18 min. The lysate was serially diluted at 1:10 and 10 μl from each well was plated on LB agar plates. LB plates were incubated overnight at 37°C, followed by CFU enumeration. The procedure was repeated with THP-1 cells with a 24-h pre-treatment and PMA differentiation, as described above. Experiments were performed with three independent runs of three replicates per run.

### 2.6. Measuring eukaryotic translational fidelity

To measure the stop codon readthrough, a stop codon was inserted between Rluc and Fluc (Fujii et al., 2018). The constructs monitoring translation error were transfected to mESC in 24-well plates using Lipofectamine 2000 according to the manufacturer’s protocol (Invitrogen). The transfected cells were treated with aminoglycoside antibiotics (gentamicin and paromomycin) for 6 h post-transfection, followed by an 18 h incubation, while the experimental group was treated with 3.1 μg/mL AgNPs for 18 h. For the combination experiment, AgNPs were mixed in the presence of aminoglycosides (amikacin and gentamicin) at concentrations indicated in the figure legend. Cells were washed with PBS and samples were harvested by Passive Lysis Buffer and assayed using the Dual-Luciferase kit (Promega). To measure the mistranslation rate, the input amount was normalized by measuring the ratio between Fluc/Rluc activity and normalized to a no-treatment control. The translation error rate was expressed as the relative ratio of Fluc to Rluc, which represent the fidelity of protein synthesis.

### 2.7. Paraformaldehyde killing of *Escherichia coli* OP50

Paraformaldehyde (PFA) killing of *E. coli* OP50 was performed as described previously (Beydoun et al., 2021). Briefly, a 200 μl aliquot from a frozen laboratory glycerol stock of *E. coli* OP50 was added to 200 mL of LB medium, then incubated for 16 h at 37°C, shaking at 220 rpm. PFA was added to a final concentration of 0.5% v/v and was incubated for 1 hour at 37°C, shaking at 220 rpm. The culture was centrifuged at 3000 RCF for 20 min. The supernatant was removed, bacteria were washed four times with fresh LB medium, and the culture was resuspended in 20 mL of fresh LB medium. The lack of viability was confirmed by plating for CFUs.

### 2.8. Effect of AgNPs on *Caenorhabditis elegans* motility

Motility was assessed by adapting the thrashing assay described elsewhere (Sawin et al., 2000). Worms were age-synchronized to the L1 stage by bleaching (Porta-de-la-Riva et al., 2012). Briefly, worms were grown to the gravid adult stage on NGM plates seeded with *E. coli* OP50. The worms were washed with M9 minimal medium to remove the bacteria from the surface, then incubated with an alkaline hypochlorite solution until the adult tissues began dissolving. The reaction was stopped with M9, and the alkaline hypochlorite solution was washed off with M9 three times. Worms were incubated at room temperature on a nutator in M9 overnight. Synchronized worms were plated onto plain and AgNP-supplemented (0.2 and 1.0 μg/mL) NGM plates seeded with PFA-killed *E. coli* OP50, followed by a 72-h incubation. Following the incubation, a single worm was placed into a drop of M9 on a microscope slide and allowed to acclimate for 1 min. The rate of motility was recorded as the number of body bends in 30 s. A body bend was defined as an approximately 90° bend along the midline with a decrease in nose-to-tail distance. The experiment was repeated twice with a total of 40 worms per treatment.

### 2.9. Lifespan of *Caenorhabditis elegans*

L1 stage age-synchronized worms were plated onto fresh NGM seeded with live *E. coli* OP50 and cultured until the young adult stage (~42 h at 25°C). Twenty worms were picked onto NGM plates containing 100 μg/mL of 5′-fluorodeoxyuridine (FUDR), either 0, 0.2, or 1.0 μg/mL AgNPs, and a PFA-killed *E. coli* OP50 lawn. The experiment was performed using five plates (*n* = 100) per each condition. Prior to lifespan experiments, we assessed the effect of AgNPs and FUDR on *E. coli* OP50 using a method described in 2.3.

### 2.10. Transmission electron microscopy of bacterial cells

Cells treated with AgNPs were compared to control cells using TEM. *E. coli* samples were grown overnight, as previously described. Bacterial cells were incubated with water (control group), 0.2, 3.1, or 7.2 μg/mL AgNP for 6 h. Immediately after incubation, formvar–carbon square mesh grids were cleaned via glow discharge in a PELCO easiGlow Discharge Cleaning System. Ten microliters of each bacterial sample were spotted onto its respective grid and incubated at room temperature for 45–60 s. After incubation, each grid was tapped against filter paper to remove excess liquids from the grid surface. One drop of 0.5% uranyl acetate from a 0.22 μm syringe filter was placed on each grid, covered to shelter from light, and incubated for 15–30 s at room temperature. Grids were tapped against filter paper to remove excess liquids after incubation. Prepared EM grids were placed in a secured, ventilated, dark container overnight to allow the substrate to dry. All grids were imaged using the Tecnai FEI G2 Spirit 120 kV TEM.

### 2.11. Combination of AgNPs with antimicrobials

Antibiotics were diluted in LB media and 100 μl of the dilutions were added to row A of a 96-well plate and 50 μl of LB media were added to the rest of the wells. Two-fold serial dilutions were performed from rows A-G, leaving H as the media-only control. The OD_600_ of an overnight culture of *E. coli* DH5α was adjusted to 1.0 and diluted at 1:500 in LB media containing 0.4 μg/mL of AgNPs. Aliquots of 50 μL were added to each well, further diluting AgNPs and bacteria to final concentrations of 0.2 μg/mL and 1:1,000, respectively. Bacterial growth was measured via OD_600_ using the Tecan Infinite Pro 200 for 20 h at 37°C every 30 min with a 5-s orbital shake before each read. Experiments were performed with three independent runs of three replicates per run. According to the European Committee on Antimicrobial Susceptibility Testing, the fractional inhibitory concentration (FIC) index was calculated and described as antagonistic (FIC ≥2), indifferent (1 < FIC <2), additive (0.5 < FIC ≤1), or synergistic (FIC ≤ 0.5; EUCAST, 2000).

### 2.12. Statistical analysis

Student’s t-test, one-way analysis of variance (ANOVA), and Kaplan–Meier survival analyzes were used to determine statistical significance. The data were considered statistically significant when *p* < 0.05. Statistical significance was indicated by asterisks (*p < 0.05, **p < 0.01, ***p < 0.001, ****p < 0.0001). Error bars represent the standard error of the mean (SEM). All statistical analyzes were performed using GraphPad Prism version 9.4.0 for Windows (GraphPad Software).

## 3. Results

### 3.1. Characterization of AgNPs

**Figure 1:**
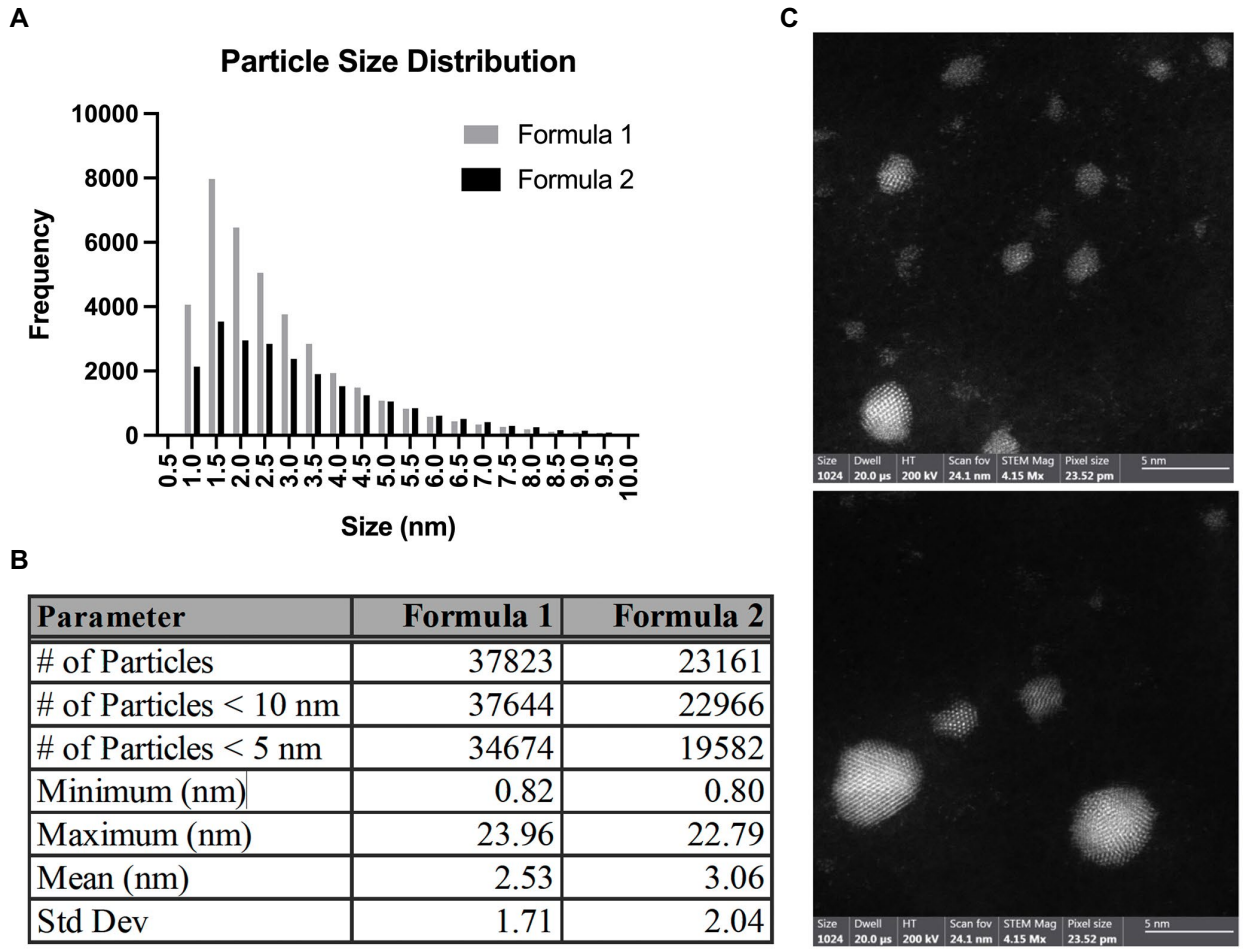
Size distribution of AgNPs. **(A)** A graph showing particle size distribution for Formula 1 and 2 AgNPs. **(B)** Measurement parameters were obtained for Formula 1 and 2 AgNP samples. **(C)** Select electron micrographs of silver nanoparticles under high-angle annular dark-field STEM mode. Scale bar = 5 nm.

While various formulations of AgNPs are available for research and public use, most, if not all of them, exhibit some level of antibacterial activity. We used commercially available AgNPs to investigate their antimicrobial properties and potential utility for future applications that could limit the need for traditional antibiotics. The average Ag concentrations of Formula 1 and Formula 2 measured by AAS were 12.3 ± 0.2 μg/mL and 26.8 ± 0.5 μg/mL, respectively (Supplementary Table S2). To determine the representative AgNP size distribution, five batches of two different formulations (Formula 1 and 2) were imaged by TEM. Two grids from each batch were imaged, and a minimum of three images from each grid were used, resulting in a robust sample size of over 37,000 and 23,000 particles from Formula 1 and 2, respectively. The resulting mean particle size in Formula 1 was 2.53 ± 1.71 and 3.06 ± 2.04 nm in Formula 2 (Figures 1A,B; Supplementary Figure S1). Size distribution analysis revealed that particles range between 0.8 and 23 nm, with over 99% of particles being < 10 nm in diameter (Figure 1B). HAADF-STEM imaging using a Cs probe-corrected FEI Themis S/TEM was used to observe the atomic structure of the AgNPs (Figure 1C). For particles approximately 2 nm and larger, a clear crystalline lattice structure was evident. Additionally, measurement of the spacing of the atomic planes (d spacing) in the AgNPs revealed a commonly observed d spacing of 0.24 ± 0.01 nm. This measured d spacing is basically identical to the largest observable d spacing in elemental Ag of 0.24 nm, corresponding to the {111} family of planes. Thus, the d spacing measurements obtained from HAADF-STEM imaging support that the particles are indeed elemental Ag. For particles smaller than 2 nm, the clear lattice structure was no longer detected (Figure 1C). We used atomic absorption spectroscopy to measure the Ag concentration of different batches within the two formulations. We found that the average Ag concentration of Formula 1 was 12.3 μg/mL while Formula 2 was 26.8 μg/mL (Supplementary Table S2).

### 3.2. Antimicrobial efficacy

**Figure 2:**
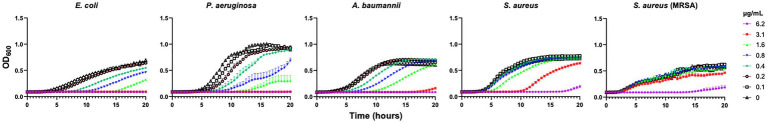
AgNPs broadly inhibit the growth of bacteria. Growth of *E. coli* DH5α, *P. aeruginosa* PAO1, *A. baumannii* 17978, *S. aureus* 12600 and *S. aureus* MRSA 131 after incubation with 0–6.2 μg/mL AgNP Formula 1.1 was assessed by measuring OD_600_ for 20 h at 37°C. Each growth curve is an average of three independent experiments with three internal replicates per run. Error bars represent SEM.

Next, we tested the antibacterial property of five batches from each formulation. To accomplish this, we initially measured the MICs of AgNPs using *E. coli* DH5α. We found comparable MICs of ~3 μg/mL using all samples (Supplementary Table S2), indicating a consistent antimicrobial property of AgNPs across all five batches from each formulation tested. Because of the similar MICs, we chose only three formulations (Formula 1.1, 2.1, and 2.2) to test the antimicrobial activity against additional bacteria, including *P. aeruginosa*, *A. baumannii*, and methicillin-resistant and susceptible *S. aureus*. Bacterial growth was inhibited in a dose-dependent manner (Figure 2), and all formulations tested were between 2 and 3 times more effective against gram-negative strains compared to gram-positive *S. aureus* strains (Supplementary Table S3). Among the three samples tested, Formula 1.1 was the most effective against all bacteria, especially the gram-negative; as such, we chose this formulation for all future experiments. Additionally, Formula 1 was generally more effective than Formula 2, a result that could be attributed to the particle size and which would be in agreement with a known correlation between particle size and antimicrobial efficacy (Morones et al., 2005; Bin Ahmad et al., 2012; Raza et al., 2016; Dong et al., 2019).

**Figure 3:**
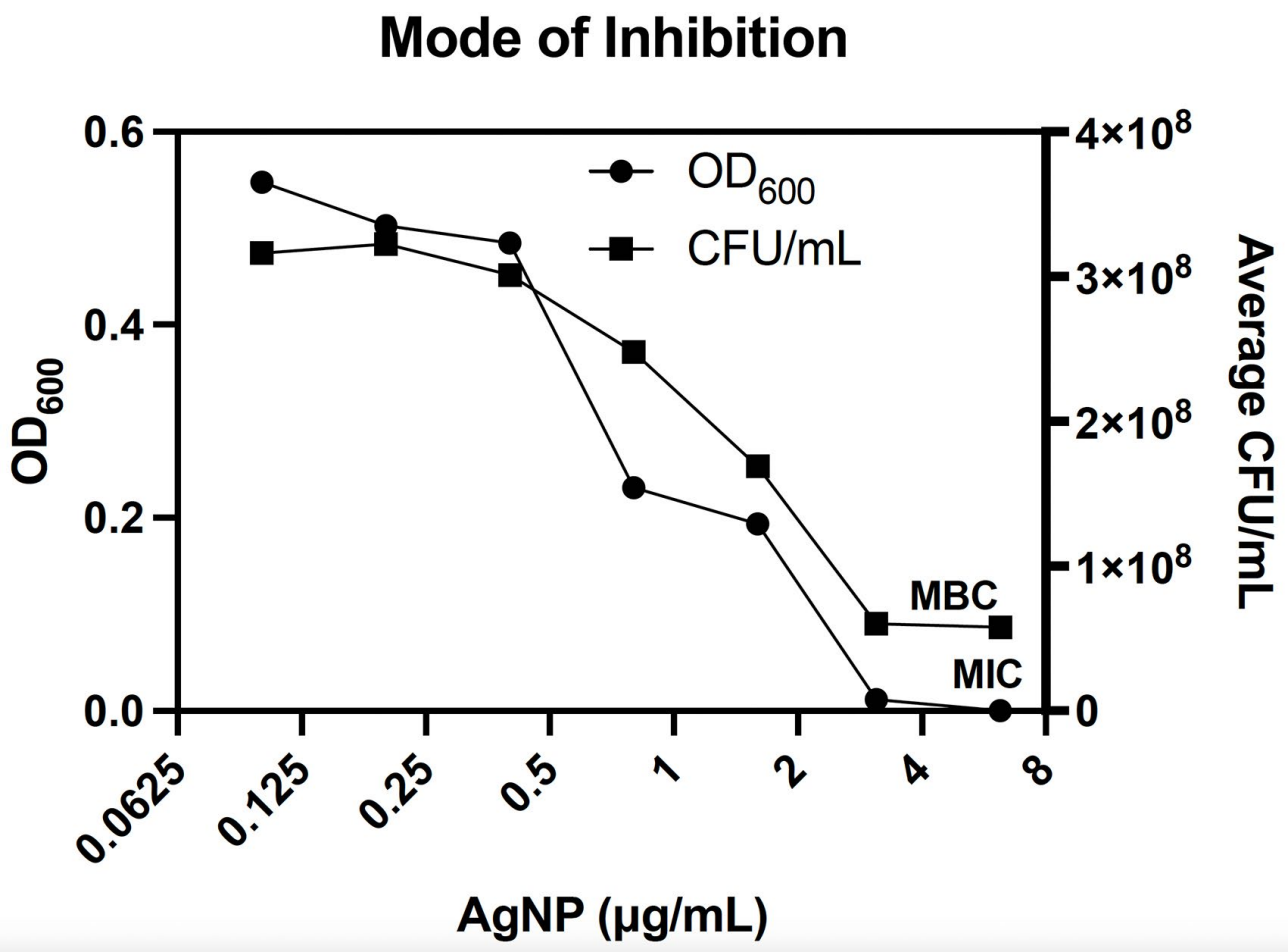
AgNPs are bactericidal against *E. coli*. The minimum bactericidal concentration (MBC) of Formula 1.1 AgNPs for *E. coli* DH5α was 6.2 μg/mL, while the MIC was 3.1 μg/mL. The ratio between the two values is 2, indicating a bactericidal mode of inhibition. Bacterial growth was assessed by measuring OD_600_ for 20 h, followed by plating for CFUs. The MIC and MBC points are marked on the graph. Data represent three independent experiments with three internal replicates per run.

To determine whether AgNPs are also effective against antibiotic-resistant bacteria, we used isolates from the CDC Antimicrobial Resistance Isolate Bank, specifically from the New or Novel Antibiotic Resistance panel. Notably, many bacterial strains within this panel carry mobile colistin resistance (mcr) genes that confer resistance to colistin by modifying the bacterial cell membrane (Liu et al., 2016; Li et al., 2020). Hyperlinks to the corresponding antibiograms for each strain can be found in Supplementary Table S1. Given that AgNPs are thought to target the bacterial membrane, these bacteria represent good candidates for antimicrobial tests (Bondarenko et al., 2018). Colistin is also classified as a last-resort polymyxin antibiotic used against gram-negative aerobic bacteria that are common etiological agents of skin infections (Levin et al., 1999; Garnacho-Montero et al., 2003; Michalopoulos et al., 2005). While the antimicrobial property of AgNPs differed substantially against bacteria with various antibiotic resistance mechanisms, the overall low MIC values that range from 3.1 to 5.5 μg/mL, except for some *Salmonella* spp., emphasize excellent antibacterial properties against broad antibiotic-resistant bacteria (Figure 2; Table 1). Among the bacteria tested, *Salmonella* spp. seem to be the least sensitive to AgNPs. Next, we assessed the mode of inhibition. It is generally accepted that if the ratio between the minimum bactericidal concentration (MBC) and the MIC is >4, then the mode of inhibition is bacteriostatic (Pankey and Sabath, 2004). We used *E. coli* DH5α to determine the MIC by monitoring OD_600_ in the presence of AgNPs over 20 h. The MBC was determined by enumerating the bacteria at the end of the treatment using the CFU plating method. The MIC was 3.125 μg/mL, and the MBC was 6.2 μg/mL, resulting in a ratio of ~2, thus, revealing a bactericidal mode of killing (Figure 3).

**Table 1:** AgNPs have antibacterial activity against clinical isolates with new and emerging resistance genes.

Bacteria	AR bank number	Novel resistance genes	MIC (μg/mL)
*E. coli*	0346	*aadA5, catA1, bla_CMY-2_, bla_CTX-M-55_, dfrA17, fosA, mcr-1, mph(A), rmtB, strA, strB, sul1, sul2*	5.2 ± 1.6
*E. coli*	0348	*bla_CMY-2_, dfrA1, ompF, strA, strB, sul2, bla_TEM-1B_*	5.2 ± 1.6
*E. coli*	0349	*mcr-1*	3.1 ± 0.0
*E. coli*	0350	*mcr-1*	3.1 ± 0.0
*E. coli*	0493	*mcr-1*	3.1 ± 0.0
*E. coli*	0494	*mcr-1*	3.1 ± 0.0
*E. coli*	0495	*mcr-1*	3.1 ± 0.0
*S.* Enteritidis	0496	*mcr-1*	5.2 ± 1.6
*S.* Typhimurium	0539	*mcr-3*	≥6.2
*S.* Typhimurium	0635	*mcr-4*	≥6.2
*S.* Oslo	0540	*mcr-3*	≥6.2
*K. pneumoniae*	0497	*mcr-1*	5.5 ± 1.4
*C. freundii*	0637	*bla_CMY-48_*	3.1 ± 0.0

### 3.3. Assessment of AgNP toxicity

#### 3.3.1. Tissue culture

**Figure 4:**
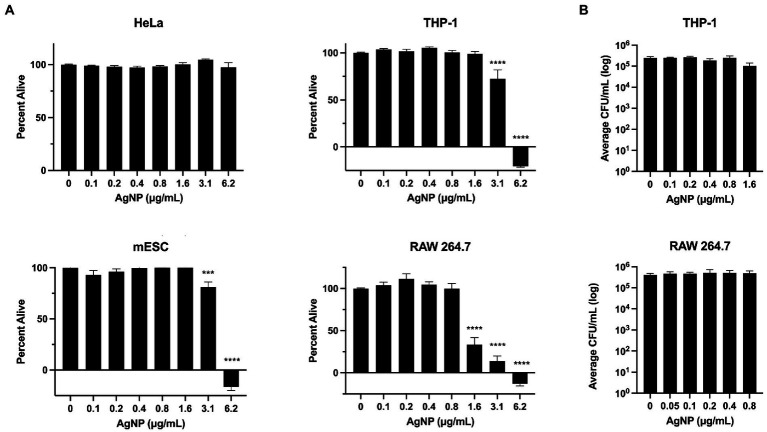
AgNP cytotoxicity assessed using different cell types. **(A)** The assessment of AgNP cytotoxicity to HeLa, pre-treated for 24 h, mESC, pre-treated for 18 h, THP-1, pre-treated for 24 h, and RAW 264.7 cells, pre-treated for 4 h, reveals cell type-specificity. **(B)** The effect of AgNPs on bacterial uptake by phagocytic macrophages at non-cytotoxic concentrations. Error bars represent SEM. Percent alive values were normalized to the control. Statistical significance was calculated using a one-way analysis of variance (ANOVA) followed by multiple comparison Dunnett’s post-hoc test (***p < 0.0005, ****p < 0.0001). Error bars represent SEM. Data represent three independent experiments with three internal replicates per run.

To better understand the effect of AgNPs on various biological systems, we employed *in vitro* and *in vivo* models to assess cytotoxicity. We measured the effect of AgNPs on the viability of mouse RAW 264.7 and human THP-1 macrophages, human epithelial HeLa cells, and mouse embryonic stem cells, mESCs. While the epithelial cells were not affected by any of the tested concentrations, the viability of macrophages and mESCs was affected at higher AgNP concentrations; however, no cytotoxicity was detected at lower bactericidal concentrations (<1.6 μg/mL), which already exhibit moderate antibacterial effects at sub-MIC levels (Figures 2, 4A). Previous reports suggest that Ag may target the cell membrane, which in eukaryotic cells could potentially impair the ability of phagocytes to uptake bacteria. To determine the effect of Ag on macrophage-mediated bacterial uptake, we pre-treated cells for 4 or 24 h with non-toxic AgNP concentrations. We found that bacterial uptake was not affected at the concentrations tested (Figure 4B).

#### 3.3.2. Proteotoxicity

**Figure 5:**
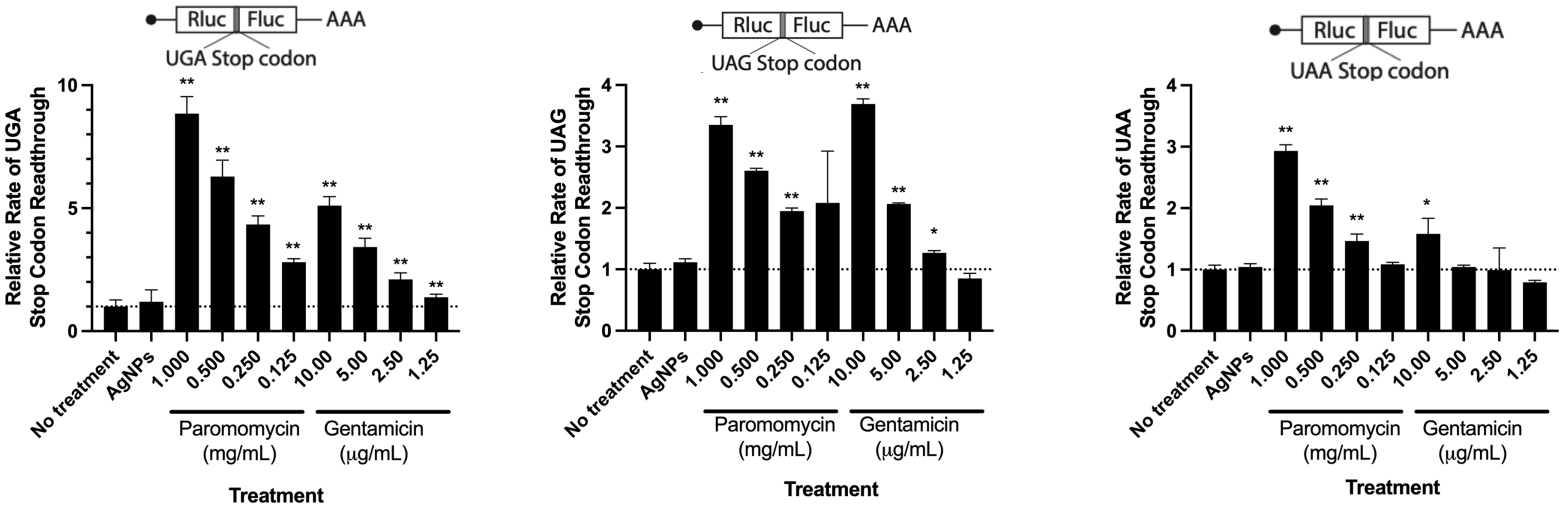
The effect of AgNPs on translation fidelity. Bar graphs representing relative rates of readthrough three stop codons: UGA (left), UAG (middle), and UAA (right). Dose–response concentrations of aminoglycosides paromomycin and gentamicin were used as positive controls. Each bar is an average of a minimum of three biological replicates. Error bars represent standard deviation (StDev). Statistical significance was calculated using the Student t-test (*p < 0.05, **p < 0.01).

Previous research suggests that the antibacterial property of Ag is mediated by its ability to target and denature the ribosome (Yamanaka et al., 2005). Similarly, it has been shown that aminoglycoside antibiotics induce misreading of mRNA by bacterial and eukaryotic ribosomes and ultimately lead to translation error which is likely the source of the proteotoxic stress (Krause et al., 2016; Prokhorova et al., 2017; Wohlgemuth et al., 2021). To test whether AgNPs could also induce misreading of mRNA in host cells, we monitored translation errors using reporters that are based on a tandem renilla luciferase (Rluc) and firefly luciferase (Fluc) linked together with a region containing a stop codon. We used three different constructs, each with a different stop codon (UGA, UAG, or UAA). The stop codon readthrough was monitored by Fluc activity. The mistranslation rate was measured by calculating the ratio between Fluc and Rluc. While we saw a significant increase in mistranslation in the presence of paromomycin and gentamicin, two aminoglycoside antibiotics that kill bacteria by inducing mistranslation (Davis, 1987; Vicens and Westhof, 2001; Kohanski et al., 2008; Krause et al., 2016), we did not detect any changes in the presence of AgNPs. We assessed the stop codon-readthrough in the presence of *E. coli* MIC (3.1 μg/mL) concentration of AgNPs. Although this concentration slightly affected the viability of mESCs, we did not detect any significant effect on translation fidelity (Figure 4A), indicating that the particles are not proteotoxic to mammalian cells under the tested conditions (Figure 5).

#### 3.3.3. Caenorhabditis elegans

We measured the effects of AgNPs on *C. elegans* motility and lifespan. The *C. elegans* model has been routinely used to assess the cytotoxicity of compounds, including AgNPs (Roh et al., 2012; Kim et al., 2017; Luo et al., 2017); as such, this model will allow us to gain further insights into the safety of AgNPs. All tests with *C. elegans* were performed using control non-treated worms or in the presence of 0.2 and 1.0 μg/mL of AgNPs supplemented into NGM seeded with an *E. coli* OP50 bacterial lawn as food. We chose 0.2 μg/mL because this low concentration exhibited a minimal antimicrobial effect in the axenic medium and was non-toxic in tissue culture while 1.0 μg/mL had an intermediate antimicrobial effect (Figure 2). To avoid crowding during lifespan measurements, we supplemented NGM plates with 5-fluoro-2′-deoxyuridine (FUDR). We found that *E. coli* OP50 growth is affected by AgNPs and FUDR (Supplementary Figure S2); therefore, to ensure that bacterial viability will not interfere with the results, we performed motility and lifespan experiments with worms that were fed pre-killed *E. coli* OP50.

**Figure 6:**
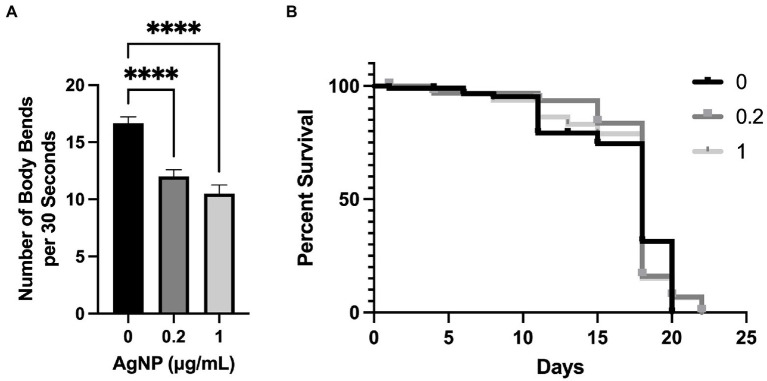
The effect of AgNPs on *C. elegans* motility and lifespan. **(A)** *C. elegans* motility was assessed in the presence of 0.2 and 1.0 μg/mL of AgNPs and a non-treated control by measuring the number of body bends (thrashing) per 30 s in a total of 40 worms per each group. **(B)** *C. elegans* lifespan was assessed in non-treated control worms and in the presence of 0.2 and 1.0 μg/mL of AgNPs using a total of 100 worms per group. Error bars represent SEM. Statistical significance was calculated using a one-way analysis of variance (ANOVA) followed by multiple comparison Dunnett’s post-hoc test (****p < 0.0001) for motility and the Kaplan–Meier survival analysis for lifespan. The difference in lifespan between the control and 0.2 and 1.0 μg/mL AgNPs was non-significant.

To evaluate the effect of AgNPs on *C. elegans* motility, worms were age-synchronized and plated for 72 h on NGM containing AgNPs and dead *E. coli* OP50 as a food source. We performed a thrashing assay, a commonly used physiological assessment of neuromuscular function (Brignull et al., 2006; Contreras et al., 2014; Zhang et al., 2021). The assay is performed by counting the number of body bends the worm performs in a 30-s interval when placed in a drop of liquid. The worms on the AgNP-containing plates performed significantly fewer body bends compared to the control (Figure 6A). However, at the same concentrations, AgNPs did not seem to have adverse effects on lifespan as we found no significant change between the control group and *C. elegans* cultured on AgNPs (Figure 6B).

### 3.4. Bacterial morphology

**Figure 7:**
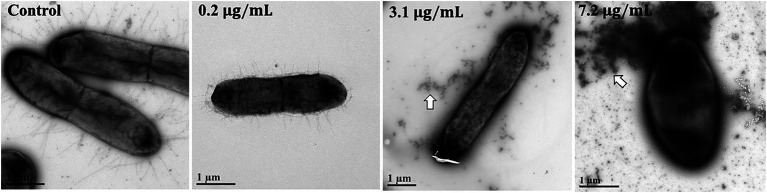
TEM images of AgNP-treated *E. coli* cells. *E. coli* cells were incubated for 6 h with (left to right) H_2_O control, 0.2 μg/mL, 3.1 μg/mL, and 7.2 μg/mL. The samples were immediately fixed to Formvar/carbon grids. Each grid was stained for 20 s with 0.5% uranyl acetate and imaged with Tecnai G2 Spirit TWIN 120 kV TEM. While arrows point to the observed dark staining of the extracellular matrix. Scale bars = 1 μm.

To determine whether AgNPs induce any visible morphological changes in bacterial cells, we employed TEM to image non-treated *E. coli* and cells treated with 0.2 μg/mL, 3.1 μg/mL, and 7.2 μg/mL for 6 h. While we did not observe any obvious morphological changes in cells incubated with 0.2 and 3.1 μg/mL AgNPs, we did observe some changes at 7.2 μg/mL. Interestingly, dark staining of the extracellular matrix seems to increase with AgNP concentration. These dark regions indicate high electron density, suggesting Ag may bind bacterial fimbriae (Figure 7). Moreover, the cells incubated with the highest concentration of AgNPs seem to be darker, which could either indicate an increased Ag concentration or cell permeability which increases uranyl acetate staining. These results warrant further experimentation to confirm fimbriae binding and its possible physiological relevance.

### 3.5. AgNPs as a potential adjuvant to antimicrobials

**Figure 8:**
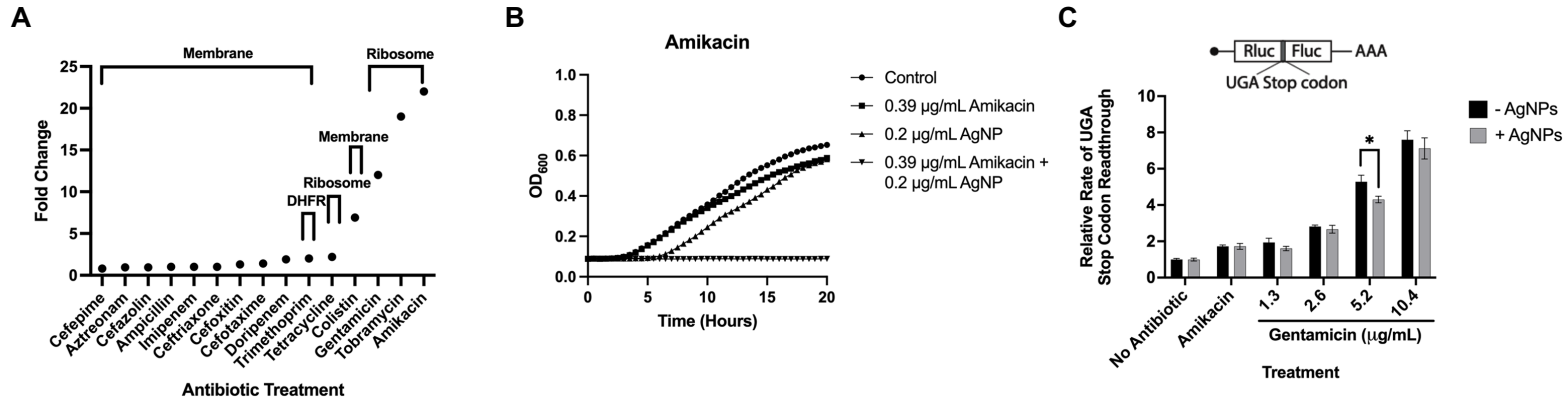
Antimicrobial efficacy of AgNPs in combination with antibiotics. **(A)** A graph representing the enhancement of *E. coli* DH5α inhibition (Fold Change) upon treatment with 0.2 μg/mL AgNPs and various combinations of antibiotics that target the bacterial membrane, DHFR, and the ribosome. **(B)** *E. coli* DH5α growth curves assessing the antimicrobial effect of AgNPs alone or in combination with amikacin. Data represent the average of three independent runs with three replicates each. **(C)** Bar graphs representing relative rates of readthrough the UGA stop codon in the absence (− AgNPs) and presence (+ AgNPs) of nanoparticles in combination with aminoglycoside antibiotics, amikacin (10.4 μg/mL) and gentamicin (1.3–10.4 μg/mL). Each bar is an average of a minimum of three biological replicates. Error bars represent StDev.

**Figure 9:**
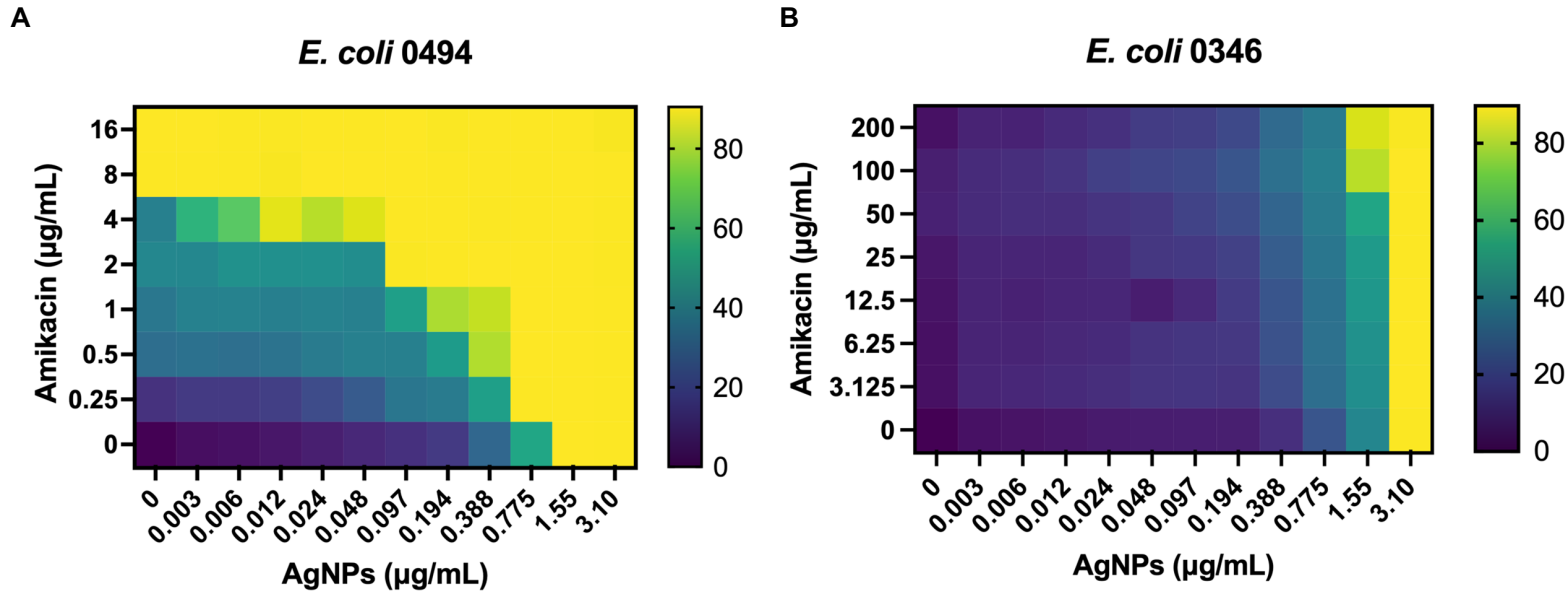
Checkerboard analysis of AgNP and amikacin combination against MDR *E. coli*. A dose–response combination of AgNPs with amikacin was tested against **(A)** *E. coli* 0494, amikacin-sensitive strain and **(B)** *E. coli* 0346, amikacin-resistant strain. The checkerboard represents an average of three independent experiments. The FICI for the amikacin-sensitive strain indicates a partial synergy. See Methods 2.3 for details. FICI = FIC_AgNP_ + FIC_Amikacin_; FIC_AgNP_ = MIC_AgNP_ combination/MIC_AgNP_ alone; FIC_Amikacin_ = MIC_Amikacin_ combination/MIC_Amikacin_ alone. ΣFICI = (0.755/1.55) + (0.25/8.0) = 0.52.

Ag is thought to have multiple cellular targets, including the bacterial membrane, DNA, and ribosome, which are also common antibiotic targets (Dakal et al., 2016). To determine the antimicrobial efficacy of AgNPs in combination with antibiotics having potentially overlapping targets, we chose several antimicrobials with various mechanisms of action that target the membrane, outer membrane, 30S ribosome, 50S ribosome, and dihydrofolate reductase. We used *E. coli* DH5α to test these antibiotics in combination with 0.2 μg/mL AgNPs, a concentration with a minimal effect (Figure 2). We assessed the effect of the combination by measuring the MIC fold-change as the ratio between the MIC of the antibiotic alone and the MIC of the combination (Table 2; Figure 8A). There was little effect on the MIC when AgNPs were tested in combination with antimicrobials that target the membrane. The only exception to this was colistin, which had a 6.9-fold decrease in the MIC. Trimethoprim, which targets dihydrofolate reductase (DHFR), had a 2-fold decrease in the MIC (Table 2; Figure 8A). When the AgNPs were combined with ribosome-targeting antibiotics, specifically aminoglycosides, the MICs decreased by up to 22-fold. The strongest synergy between AgNPs and antibiotics was detected with amikacin, an aminoglycoside that targets the 30S ribosome, which resulted in a 22-fold decrease in the MIC. When amikacin and AgNPs were combined at their non-effective concentrations of 0.39 and 0.2 μg/mL, respectively, *E. coli* DH5α was completely inhibited, indicating a strong synergy between the two (Table 2; Figure 8B). Surprisingly, when we tested the combination with tetracycline, which also targets the 30S ribosomal subunit, we did not observe any synergistic effect. We have also ruled out any contribution from the potential inhibition by the solvents, DMSO, ethanol, or water at the highest tested dilutions (Supplementary Figure S3). We thought that perhaps the synergistic effect between AgNPs and aminoglycosides might also affect the host cells by inducing translation errors; however, after testing the combination, we found no effect on the translation error in the host cells (Figure 8C). Further testing of an AgNP dose–response combination with amikacin against MDR *E. coli* strains using the checkerboard assay also revealed an antimicrobial enhancement (Figure 9). The 0.52 FICI value of the combination treatment used against the MDR, but amikacin-sensitive strain (Figure 9A), indicates a partial synergy. We were not able to calculate the FICI value for the amikacin-resistant strain. These results further support the AgNP-mediated enhancement of antimicrobial therapies. Collectively, the antimicrobial efficacy of AgNPs and aminoglycosides offers a promising combination treatment strategy for antibiotic-resistant bacteria.

**Table 2:** AgNPs synergize with antibiotics against *E. coli*.

Treatment	Target	MIC (μg/mL)	MIC of antibiotic (μg/mL) + AgNPs (0.2 μg/mL)	Fold change
AgNPs		3.1 ± 0.0		
Cefepime	Membrane	0.040 ± 0.000	0.053 ± 0.020	0.80
Aztreonam	Membrane	0.076 ± 0.026	0.081 ± 0.024	0.94
Cefazolin	Membrane	1.7 ± 0.6	1.8 ± 0.66	0.94
Ampicillin	Membrane	31 ± 11	31 ± 11	1.0
Imipenem	Membrane	≥12.5	≥12.5	1.0
Ceftriaxone	Membrane	0.031 ± 0.000	0.030 ± 0.005	1.0
Cefoxitin	Membrane	3.5 ± 1.0	2.8 ± 0.69	1.3
Cefotaxime	Membrane	0.026 ± 0.028	0.018 ± 0.013	1.4
Doripenem	Membrane	0.045 ± 0.032	0.024 ± 0.008	1.9
Trimethoprim	DHFR	≥50	≥25	2.0
Tetracycline	30S Ribosome	0.83 ± 0.26	0.38 ± 0.14	2.2
Colistin	Outer Membrane	0.25 ± 0.00	0.036 ± 0.021	6.9
Gentamicin	30S Ribosome	4.5 ± 1.6	0.39 ± 0.00	12
Tobramycin	30S and 50S Ribosome	3.8 ± 1.9	0.20 ± 0.00	19
Amikacin	30S Ribosome	8.7 ± 4.6	0.39 ± 0.00	22

## 4. Discussion

In the present study, we assessed the antimicrobial efficacy and cytotoxicity of AgNPs. Physical characterization of these particles revealed a high consistency in size between the five different batches from each formulation. We observed a slight but significant difference in MIC values that positively correlated with the average size of the particles (Supplementary Table S2). The size-antimicrobial efficacy correlation of AgNPs is well established (Morones et al., 2005; Bin Ahmad et al., 2012; Raza et al., 2016; Dong et al., 2019); therefore, our results are in agreement with previous reports. The *E. coli* DH5α MICs of our formulations were in the ~3 μg/mL range, which is lower than other AgNP formulations (Lara et al., 2010) but higher than Ag ions alone (Jung et al., 2008; Li et al., 2017). Although, it is difficult to directly compare the results between studies because of the differences in the experimental design, antimicrobial metrics, and size of the particles. The low variability in the size and antibacterial efficacy emphasize the reproducibility, consistency, and stability of our AgNP formulations. Interestingly, we found that AgNPs are more effective against gram-negative bacteria, a result that was previously observed (Kim et al., 2007; Jung et al., 2008). While the mechanisms contributing to this difference are not known, it is thought that perhaps the dissociated Ag ions may more readily enter gram-negative bacteria via outer membrane porins (Radzig et al., 2013). Other reports suggest that the thickness of the peptidoglycan layer is responsible for the difference in AgNP efficacy between gram-positive and gram-negative bacteria (Dakal et al., 2016). Regardless of the mechanisms responsible for the difference, the potent antimicrobial property against gram-negative bacteria is promising as these organisms are notoriously known for their intrinsic resistance to many classes of antibiotics, leaving little to no treatment options available (Breijyeh et al., 2020). Our results show that AgNPs inhibit the growth of a broad spectrum of gram-negative bacteria with novel resistance mechanisms, including strains resistant to colistin, a last-resort highly toxic antibiotic reserved for MDR gram-negative infections (El-Sayed Ahmed et al., 2020). The low MICs (<6.2 μg/mL) against *Escherichia*, *Pseudomonas*, *Acinetobacter*, *Klebsiella*, *Citrobacter*, and *Salmonella* species, indicate a promising antimicrobial. Others have reported similar MIC values for various gram-negative bacteria, including *E. coli*, *K. pneumoniae*, *P. aeruginosa*, and *S. aureus* (Liao et al., 2019; Desouky et al., 2020; Mohamed et al., 2020). Collectively, the nanoparticle formulation used in this study is consistent with the antimicrobial properties of other AgNPs.

While Ag is known for its robust antimicrobial property, it also exhibits cytotoxicity, which could limit its application as a broad oral antimicrobial. We employed various methods to assess the cytotoxic effect of AgNPs on living systems and found little to no toxicity at lower bactericidal concentrations when applied in a tissue-culture model (Figure 4). We noted that phagocytic cells were more sensitive to Ag, which, based on the physiological function of these cells, could be the result of enhanced particle uptake. Given the membrane-targeting property of Ag, we also tested the effect of our nanoparticles on macrophage-mediated bacterial uptake. We did not find any significant effect on bacterial uptake, which is consistent with previous results where treatment has either no effect or leads to the enhancement of phagocytosis (Haase et al., 2012; Alsaleh et al., 2019; Musino et al., 2021). Macrophages are involved in wound healing and protection against bacterial invasion; as such, demonstrating the inert effect of AgNPs on phagocytosis could also warrant their possible use as an oral antimicrobial. Although, topical applications, such as burn, surgical and suture wounds, dental applications, and eye, ear, and skin infections, would allow using higher concentrations without the risk of adverse reactions, which is often the case with topical antibiotics. Based on the previous reports suggesting that Ag targets the bacterial ribosome, we wanted to investigate whether AgNPs may also affect the eukaryotic ribosome; such cross-targeting is seen with aminoglycosides (Hobbie et al., 2008; Hong et al., 2015; Prokhorova et al., 2017). We did not detect any effect of AgNPs on translation fidelity under our specific test conditions. However, we do not eliminate the possibility that AgNPs still target the eukaryotic ribosome without affecting the mistranslation rate.

Numerous reports have assessed the efficacy and cytotoxicity of AgNPs. However, the differences in experimental methods and AgNP properties make it difficult to directly compare the results between studies. While the results obtained in our study are unique to our AgNP formulations, our data are vastly supported by other reports. For example, Luo et al. demonstrated that low concentration (<1 μg/mL) of AgNPs did not affect *C. elegans* lifespan when worms were cultured on NGM agar plates (Luo et al., 2017). Smaller-size AgNPs, 2 and 5 nm, did not affect *C. elegans* lifespan even at 100 μg/mL; however, 10 nm particles shortened lifespan, suggesting a less-invasive property of smaller particles (Contreras et al., 2014). However, the size-toxicity correlation is not always clear, suggesting that other AgNP properties contribute to cytotoxicity. For example, smaller-size particles (25 nm) were more toxic to the *C. elegans* germline and consequently decreased brood size compared to larger particles (75 nm). Similarly, 25 μg/ml of 25 nm AgNPs shortened *C. elegans* lifespan, while at the same concentration, 75 nm particles had no effect (Luo et al., 2016). The differences in the cytotoxicity of AgNPs are likely due to their physical and chemical properties, such as Ag concentration, size, formulation, charge, and purity, among others. Our results demonstrate that the concentrations of 0.2 and 1 μg/mL AgNPs have no effect on *C. elegans* lifespan, which agrees with the data reported across other studies. Furthermore, our data indicate that AgNPs affect *C. elegans* motility (Figure 6A), a result that has also been observed by others (Contreras et al., 2014). Since the smaller particles could more readily penetrate cells, it is possible that muscle is more sensitive than other tissues; however, it remains to be determined whether this is a worm-specific result or whether these results could be translatable to higher eukaryotes, mammalian models, and humans. In general, the results of our experiments warrant further studies into the applicability of AgNPs as topical antimicrobials.

Our TEM results suggest that AgNPs bind to bacterial fimbriae. If this is the case, then one would expect detectable effects on bacterial attachment and biofilm formation. Given that fimbriae support bacterial virulent mechanisms (Spurbeck et al., 2011; Sarowska et al., 2019), targeting these structures could provide a novel approach to inhibit bacterial pathogenicity. In fact, the anti-biofilm properties of AgNPs were also observed in other studies (Mohanta et al., 2020; Siddique et al., 2020). One of the most promising properties of Ag is its ability to target antibiotic resistance mechanisms and potentiate the efficacy of antibiotics (Lara et al., 2010; Brown et al., 2012; Panáček et al., 2016; Mohamed et al., 2020; Malawong et al., 2021). Interestingly, out of the different classes of antibiotics we tested, only a combination with aminoglycosides showed synergy in the presence of AgNPs, suggesting that either Ag promotes aminoglycoside binding to the ribosome or increases intracellular aminoglycoside concentration by permeating the membrane. If the latter is the case, we should expect a robust MIC fold change in combination with other non-ribosomal-targeting antibiotics (i.e., trimethoprim), but we did not see such a synergistic effect. Additionally, a combination of AgNPs with tetracycline, which also targets the 30S ribosomal subunit, did not result in synergy; therefore, the AgNP-mediated enhancement of antibiotics is not explained by permeability, but it is more likely that AgNPs and aminoglycosides share common targets and interact synergistically at specific sites of the bacterial ribosome. We did not detect any effect of AgNPs on the fidelity of protein synthesis in mammalian cells; however, this does not eliminate the possibility of AgNPs targeting the eukaryotic ribosome without affecting its function. Furthermore, our results do not eliminate the possibility of AgNPs targeting the prokaryotic ribosome and resulting in the observed synergistic effect. Collectively, our experiments reveal a promising antibacterial activity of AgNPs and their ability to lower the MICs of aminoglycoside antibiotics. While our results emphasize the importance of choosing the right antibiotic when combining with AgNPs, further work is needed to determine the underlying mechanisms and *in vivo* efficacy.

## Data availability statement

The original contributions presented in the study are included in the article/Supplementary material, further inquiries can be directed to the corresponding author.

## Author contributions

DC: conceptualization and supervision. DC, NQ, NR, and KF: methodology. AD and DC: software, manuscript draft preparation, and visualization. AD, DC, NR, and KF: validation. AD, DD, WD, NQ, CN, LA, GE, JB, NR, KF, and DC: investigation. AD, DD, WD, NQ, GE, JB, NR, KF, and DC: manuscript review and editing. All authors contributed to the article and approved the submitted version.

## Funding

Funding for this study was provided by Natural Immunogenics Corporation and the University of Florida Industry Partnerships Grant Program awarded to DC. KF was supported by the Japanese Science and Technology Agency grant JPMJPR2049.

## Conflict of interest

NQ, CN, and LA were employed by Natural Immunogenics Corporation.

The remaining authors declare that the research was conducted in the absence of any commercial or financial relationships that could be construed as a potential conflict of interest.

The authors declare that this study received funding from Natural Immunogenics Corporation. The funder had the following involvement in the study: product characterization using AAS and TEM.

## Publisher’s note

All claims expressed in this article are solely those of the authors and do not necessarily represent those of their affiliated organizations, or those of the publisher, the editors and the reviewers. Any product that may be evaluated in this article, or claim that may be made by its manufacturer, is not guaranteed or endorsed by the publisher.
