# Supplementary material for: Silver nanoparticles enhance the efficacy of aminoglycosides against antibiotic-resistant bacteria
Source: Front Microbiol. 2023 Jan 31;13:1064095. doi: 10.3389/fmicb.2022.1064095 (PMC9927651; doi:10.3389/fmicb.2022.1064095)
Supplement: Supplementary file 1 [file Table_1.pdf]

Bacterial Strains		
**Strain**	**Source**	**Identifier**
Escherichia coli OP50	Caenorhabditis Genetics Center	WB OP50; RRID:WE-
Escherichia coli DH5a		MG040, DC146
Pseudomonas aeruginosa PAO1	Shuman Lab (University of Chicago)	PAO1; DC3
Staphylococcus aureus 12600	ATCC	ATCC 12600, DC226
Methicillin-resistant *Staphylococcus aureus* 131	BEI Resources	HM-466, MRSA 131
Acinetobacter baumannii 17978	Shuman Lab (University of Chicago)	DC2, 17978
Escherichia coli 0346	AR Isolate Bank	0346
Escherichia coli 0348	AR Isolate Bank	0348
Escherichia coli 0349	AR Isolate Bank	0349, BAA-3170
Escherichia coli 0350	AR Isolate Bank	0350
Escherichia coli 0493	AR Isolate Bank	0493
Escherichia coli 0494	AR Isolate Bank	0494
Escherichia coli 0495	AR Isolate Bank	0495
Salmonella Enteritidis 0496	AR Isolate Bank	0496
Salmonella Typhimurium 0539	AR Isolate Bank	0539
Salmonella Typhimurium 0635	AR Isolate Bank	0635
Salmonella Oslo 0540	AR Isolate Bank	0540
Klebsiella pneumoniae 0497	AR Isolate Bank	0497
Citrobacter freundii 0637	AR Isolate Bank	0637
***C. elegans* Strains**		
C. elegans N2, Bristol	Caenorhabditis Genetics Center	N2
**Cell Lines**		
THP1 monocytes	ATCC	ATCC TIB-202
RAW 264.7 macrophages	ATCC	ATCC TIB-71
HeLa	ATCC	ATCC CCL-2
Murine Embryonic Stem Cells (mESCs)	Fujii Lab (University of Florida, (Hooper et al. 1987))	E14 mESCs, male
**Chemicals**		
Cholesterol	Fisher Scientific	Cat#ICN10138201
LB (Lennox)	Apex	Cat#11-125
Potassium Phosphate Monobasic	Fisher Scientific	Cat#P285-500
BD Bacto™ Tryptic Soy Broth without Dextrose	Becton, Dickinson and Company	Cat#286220
Dulbecco's Modified Eagle Medium (DMEM)	Genesee Scientific	Cat#25-500
Roswell Park Memorial (RPMI)	Genesee Scientific	Cat#25-506
Fetal Bovine Serum (FBS)	Genesee Scientific	Cat#25-514
Phosphate Buffer Saline (PBS)	Genesee Scientific	Cat#25-507B
Bacto-Peptone	Becton, Dickinson and Company	Cat#211677
Fisher BioReagents™ Agar	Fisher Scientific	Cat#9002-18-0
Sodium Chloride (NaCl)	Fisher Scientific	Cat#7647-14-5
Calcium Chloride (CaCl2)	Fisher Chemical	Cat#79-500
Magnesium Sulfate (MgSO4)	Thermo-Alfa Aesar	Cat#10034-99-8
Potassium Phosphate (KH2PO4)	Fisher Chemical	Cat#285-500
Dimethyl Sulfoxide (DMSO)	Fisher Bioreagents	Cat#BP231-100
Amikacin	Thermo-Alfa Aesar	Cat#39831-55-5
Ampicillin	Thermo-Fisher	Cat#BP17605
Aztreonam	Tokyo Chemical Industry (TCI)	Cat#78110-38-0
Cefazolin	Chem-Impex Intl	Cat#27164-46-1
Cefepine	Chem-Impex Intl	Cat#123171-59-5
Cefotaxime	Tokyo Chemical Industry (TCI)	Cat#64485-93-4
Cefoxitin	Tokyo Chemical Industry (TCI)	Cat#35607-66-0
Ceftriaxone	Tokyo Chemical Industry (TCI)	Cat#104376-79-6
Colistin	Acros	Cat#1264-72-8
Doripenem	Thermo	Cat#148016-81-3
Gentamicin	Caisson	Cat#15750-060
Imipenem	Apex Bio	Cat#64221-86-9
Tobramycin	Acros	Cat#32986-56-4
Trimethoprim	Thermo	Cat#738-70-5
Tetracycline	Thermo-Fisher	Cat#J61714.14
Floxuridine (FUDR)	Research Products Internation (RPI)	Cat#50-91-9
Paraformaldehyde (PFA)	Thermo-Fisher	Cat#50-00-0
Cell Titer Blue Reagent	Promega	Cat#G808A
Trypsin	Corning	Cat#25-053-CI
Silver Nanoparticles (AgNPs)	Sovereign Silver	Cat#80066694
PM IF-0a GN/GP Base	Biolog	Cat#72268
PM IF-10a GN/GP Base	Biolog	Cat#72264
Biolog Redox Dye Mix A	Biolog	Cat#74221
Triton X-100 (TX-100)	Promega	Cat#H5142
Phorbol 12-myristate 13-acetate (PMA)	Thermo-Fisher	Cat#J63916
KnockOut Dulbecco's Modified Eagle Medium	Thermo-Fisher	Cat#10829018
1x L-glutamine	EMD Millipore	Cat#TMS-002-C
1x non-essential amino acids	EMD Millipore	Cat# TMS-001-C
2-mercaptoethanol	Thermo-Fisher	Cat# 034461-100
Leukimia Inhibiting Factor (LIF)	EMD Millipore	Cat#ESG1107
ES grade fetal bovine serum (FBS)	EMD Millipore	Cat#ES-009-B
ES grade penicillin/streptomycin	EMD Millipore	Cat#TMS-AB2-C
**Equipment**		
Tecan Infinite M Nano+ Microplate Reader	Tecan	Cat#30190087
Tecnai G2 Spirit TWIN Transmission Electron Microscope		
Omnilog	Biolog	Model#71000
**Software**		
GraphPad Prism v8 4.3	GraphPad Software, Inc	https://www.graphpad.com
BioRender	BioRender	www.biorender.com
Fiji	ImageJ	https://imagej.net/software/fiji/
