# Supplementary material for: Silver nanoparticles enhance the efficacy of aminoglycosides against antibiotic-resistant bacteria
Source: Front Microbiol. 2023 Jan 31;13:1064095. doi: 10.3389/fmicb.2022.1064095 (PMC9927651; doi:10.3389/fmicb.2022.1064095)
Supplement: Supplementary file 2 [file Table_2.pdf]

Formula	Silver Concentration (µg/mL)	Particle Size (nm)	MIC (µg/mL)
1.1	12.4	2.53±1.71	3.1±0
1.2	12.2	2.53±1.71	3.1±0
1.3	12.0	2.53±1.71	3.0±0
1.4	12.4	2.53±1.71	3.1±0
1.5	12.4	2.53±1.71	3.1±0
Average	12.3±0.2	2.53±1.71	3.1±0*
2.1	26.8	3.06±2.04	3.4±0
2.2	27.0	3.06±2.04	3.4±0
2.3	26.1	3.06±2.04	3.3±0
2.4	26.5	3.06±2.04	3.3±0
2.5	27.4	3.06±2.04	3.4±0
Average	26.8±0.5	3.06±2.04	3.4±0.1*

*Student t-test: $p<0.0001$
