# Supplementary material for: Silver nanoparticles enhance the efficacy of aminoglycosides against antibiotic-resistant bacteria
Source: Front Microbiol. 2023 Jan 31;13:1064095. doi: 10.3389/fmicb.2022.1064095 (PMC9927651; doi:10.3389/fmicb.2022.1064095)
Supplement: Supplementary file 3 [file Table_3.pdf]

Minimum Inhibitory Concentrations, μg/mL

**Formula**	***E. coli***	***P. aeruginosa***	***A. baumannii***	***S. aureus***	**MRSA**
1.1	3.1±0	2.8±2.0	6.2±0	≥6.2	>6.2±0
2.1	3.4±0	3.4±2.5	6.7±0	13±0	>13±0
2.2	3.4±0	3.4±2.5	6.8±0	14±0	>14±0
