# Supplementary figures and images for: Silver nanoparticles enhance the efficacy of aminoglycosides against antibiotic-resistant bacteria
Source: Front Microbiol. 2023 Jan 31;13:1064095. doi: 10.3389/fmicb.2022.1064095 (PMC9927651; doi:10.3389/fmicb.2022.1064095)

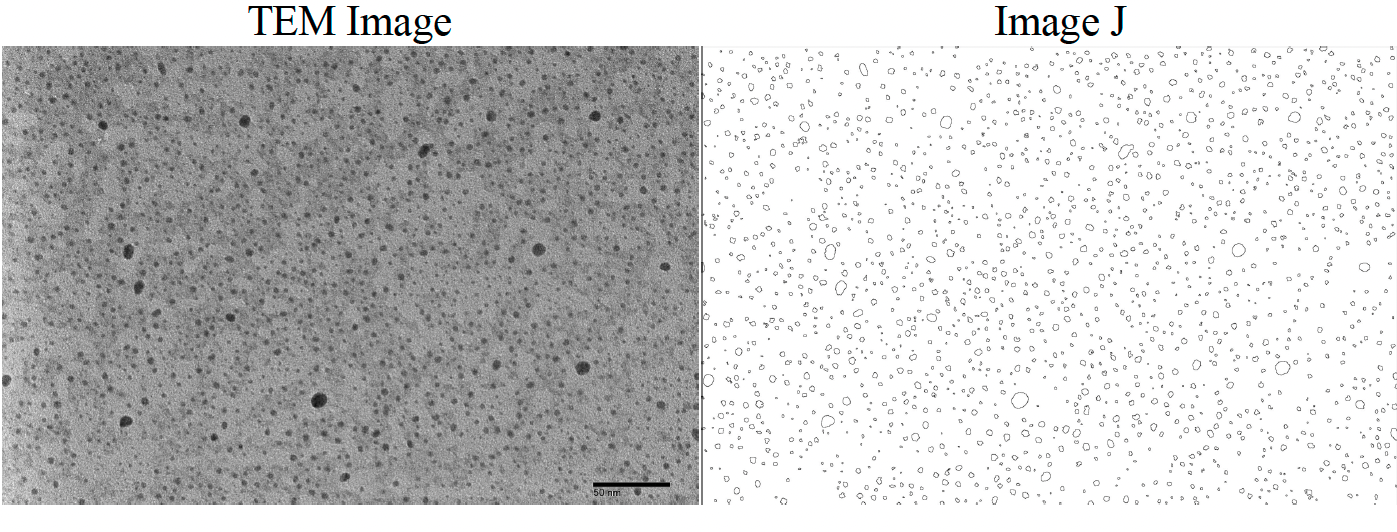

Supplement: Supplementary file 4 [file Image_1.TIFF]

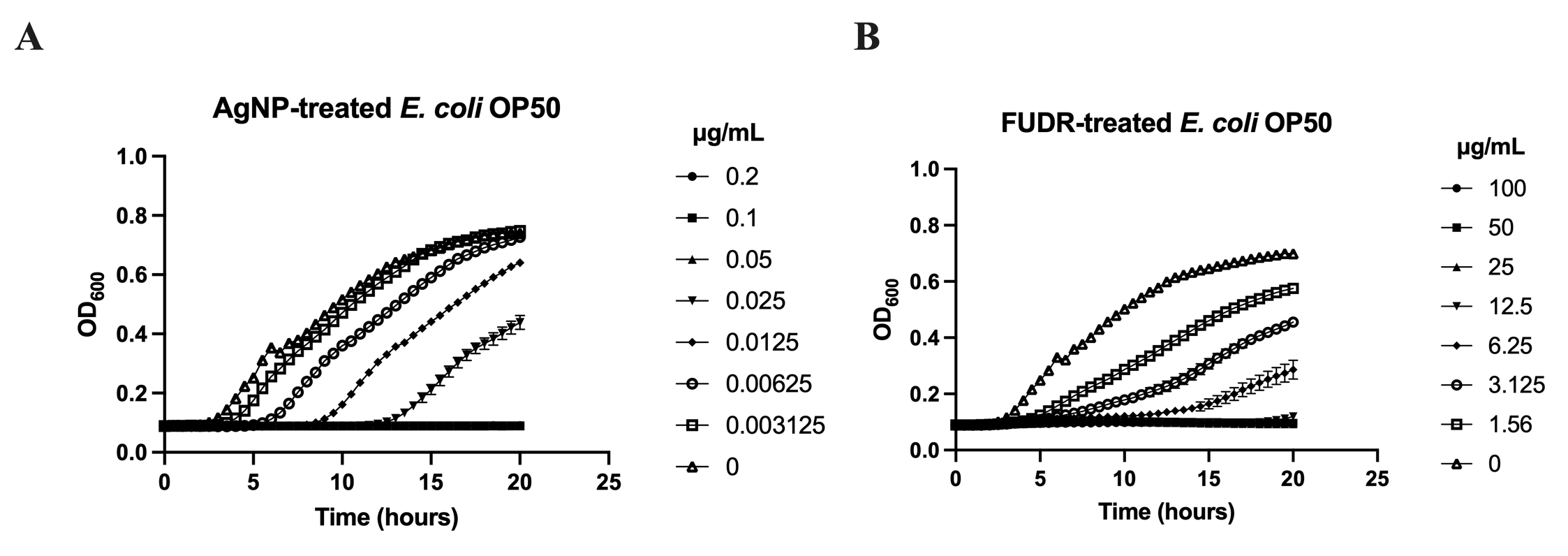

Supplement: Supplementary file 5 [file Image_2.TIFF]

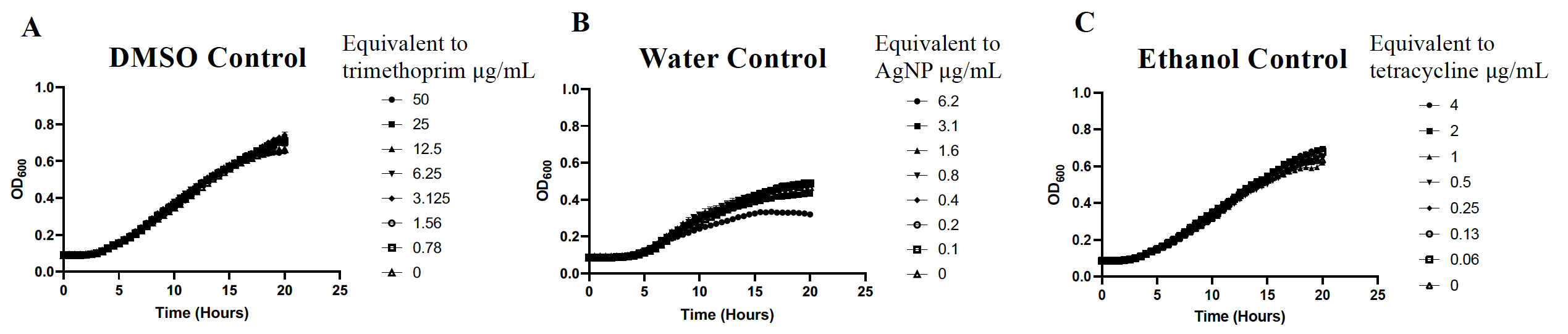

Supplement: Supplementary file 6 [file Image_3.TIFF]
